# Abnormalities in Brainstem Auditory Evoked Potentials in Sheep with Transmissible Spongiform Encephalopathies and Lack of a Clear Pathological Relationship

**DOI:** 10.3389/fvets.2016.00060

**Published:** 2016-08-02

**Authors:** Timm Konold, Laura J. Phelan, Saira Cawthraw, Marion M. Simmons, Melanie J. Chaplin, Lorenzo González

**Affiliations:** ^1^Animal Sciences Unit, Animal and Plant Health Agency, Weybridge, Addlestone, UK; ^2^Central Sequencing Unit, Animal and Plant Health Agency, Weybridge, Addlestone, UK; ^3^Pathology Department, Animal and Plant Health Agency, Weybridge, Addlestone, UK; ^4^Pathology Department, Animal and Plant Health Agency, Lasswade, Penicuik, UK

**Keywords:** brainstem auditory evoked potentials, scrapie, bovine spongiform encephalopathy, prion, vacuolation, PrP^Sc^

## Abstract

Scrapie is transmissible spongiform encephalopathy (TSE), which causes neurological signs in sheep, but confirmatory diagnosis is usually made postmortem on examination of the brain for TSE-associated markers like vacuolar changes and disease-associated prion protein (PrP^Sc^). The objective of this study was to evaluate whether testing of brainstem auditory evoked potentials (BAEPs) at two different sound levels could aid in the clinical diagnosis of TSEs in sheep naturally or experimentally infected with different TSE strains [classical and atypical scrapie and bovine spongiform encephalopathy (BSE)] and whether any BAEP abnormalities were associated with TSE-associated markers in the auditory pathways. BAEPs were recorded from 141 clinically healthy sheep of different breeds and ages that tested negative for TSEs on postmortem tests to establish a reference range and to allow comparison with 30 sheep clinically affected or exposed to classical scrapie (CS) without disease confirmation (test group 1) and 182 clinically affected sheep with disease confirmation (test group 2). Abnormal BAEPs were found in 7 sheep (23%) of group 1 and 42 sheep (23%) of group 2. The proportion of sheep with abnormalities did not appear to be influenced by TSE strain or PrP^Sc^ gene polymorphisms. When the magnitude of TSE-associated markers in the auditory pathways was compared between a subset of 12 sheep with and 12 sheep without BAEP abnormalities in group 2, no significant differences in the total PrP^Sc^ or vacuolation scores in the auditory pathways could be found. However, the data suggested that there was a difference in the PrP^Sc^ scores depending on the TSE strain because PrP^Sc^ scores were significantly higher in sheep with BAEP abnormalities infected with classical and L-type BSE, but not with CS. The results indicated that BAEPs may be abnormal in sheep infected with TSEs but the test is not specific for TSEs and that neither vacuolation nor PrP^Sc^ accumulation appears to be responsible for the clinical abnormalities.

## Introduction

Scrapie is a transmissible spongiform encephalopathy (TSE) of sheep that is characterized by a long incubation period. Two naturally occurring forms of scrapie are known to exist, classical scrapie (CS) and – more recently – atypical scrapie, which is considered to be pathologically, biochemically, biologically, and epidemiologically distinct from CS (1). Clinical signs of scrapie are usually not expected prior to neuroinvasion of the agent and are probably not evident until accumulation of disease-associated prion protein (PrP^Sc^) in the brain is already widespread because many scrapie cases that are diagnosed postmortem on examination of the brain by immunohistochemistry or other rapid tests are considered asymptomatic (2, 3). Reporting of clinical suspects is based on the display of clinical signs associated with scrapie, but these can be variable and may also include signs, such as loss of body condition and pruritus, which are equally observed in and may be confused with non-neurological diseases.

Brainstem auditory evoked potentials (BAEPs) are short-latency potentials recorded from the surface of the head during brief acoustic stimulation (4), which can be used to assess auditory function as well as various neurological disorders involving the auditory pathways in the brainstem and midbrain. BAEPs in sheep are reproducible and similar to those reported for other species (5) but have not been widely used in the neurological assessment of sheep. There are two studies that reported waveform abnormalities in ovine scrapie (6, 7), but both were based on only a small number of sheep and only one was peer-reviewed.

The auditory pathways are located in the brainstem (cochlear nuclei, dorsal olivary complex), midbrain (lateral lemniscus, caudal colliculus), thalamus (medial geniculate nucleus), and in the auditory cortex (4). Identification of the generators of the BAEP waveforms is primarily based on studies in humans and cats, which suggest that the first positive waveform (I) is generated by the vestibulocochlear nerve or spiral ganglion cells of the cochlea, the second peak (II) by cochlear nuclei, the third peak (III) by cells of the cochlear nuclei and dorsal olivary complex, and the fourth (IV) and fifth (V) peaks by neurons in dorsal olivary complex, lateral lemniscus, and caudal colliculus (4, 8, 9). Neuropathological studies of TSEs in sheep, which described the neuroanatomical distribution of vacuolation and PrP^Sc^ accumulation in the brain, demonstrated the presence of these TSE-specific markers in auditory brainstem nuclei (10, 11).

The objective of the presented study was to evaluate whether BAEP testing could aid in the clinical diagnosis of transmissible spongiform encephalopathies (TSEs) in sheep utilizing sheep naturally or experimentally infected with different TSE strains [classical and atypical scrapie and bovine spongiform encephalopathy (BSE)]. It was hypothesized that TSEs caused measurable abnormalities in BAEPs, which may be independent of TSE strain, breed, route of infection, and prion protein gene (*PRNP*) genotype. In parallel, the magnitude of pathological markers used for TSE diagnosis (vacuolation and PrP^Sc^ accumulation) was determined by neuropathological examination of neuroanatomical areas comprising the auditory pathways in selected sheep with and without BAEP abnormalities to assess whether there was a relationship between clinical and pathological findings.

## Materials and Methods

All procedures involving animals were approved under the Animal (Scientific Procedures) Act 1986 by the relevant project licenses following review by the Animal and Plant Health Agency’s animal and welfare ethical review body.

All sheep were subject to a clinical examination, either a full neurological examination as described previously (12) or a short clinical examination to detect signs of scrapie (13), prior to BAEP recording.

Genotyping of the ovine *PRNP* was carried out from blood or fresh brain according to previously described methods to determine polymorphisms at codon 112, 136, 141, 154, and 171 (14).

### Control Population

It is well known that age and gender are factors that affect BAEPs in humans (15); additional factors in animals are related to breed diversity and include weight, height, and head size (16, 17). As it was impossible to account for all these factors by establishing reference ranges for each breed, gender, and age group and a large reference dataset for sheep had not been published, the reference range was based to some extent on the recommendation by Steiss that at least 20 normal subjects (=sheep of one breed) should be studied, spanning the age range of the patients (=TSE suspects) to be examined (18).

In total, 141 sheep were examined (81 females, 7 males, and 53 neutered males) with a median age of 51 months (range 17–111) and comprised 67 Suffolk, 48 Cheviot, 22 Romney, and 4 Poll Dorset sheep. They were derived from a CS-free research flock (19), some of which were used for a range of TSE studies as environmental control sheep kept separate from infected sheep, and sheep from an experimental BSE research flock (20), which were clinically unremarkable, not experimentally challenged with any TSE strain, and tested negative for TSEs by postmortem examination (for tests, see below). Details of the sheep and their study origin are given in Table 1.

**Table 1:** **Details and origin of control sheep**.

Breed (*N*)	Sex^a^ (*N*)	Median age (range) (months)	*PRNP* (*N*)^b^	Source
Cheviot (14)	F (7)	33 (25–39)	VRQ/VRQ (14)	Controls for scrapie milk transmission experiment (21)
	M/N (7)	29 (25–39)		
Cheviot (12)	F (4)	47 (20–64)	VRQ/VRQ (2), VRQ/AHQ (1), AHQ/AHQ (1)	Experimental BSE research flock (20)
	M/N (8)	61 (50–71)	VRQ/VRQ (5), VRQ/AHQ (1), AHQ/AHQ (1), ARQ/TARQ (1)	
Cheviot (1)	M/N (1)	76	ARQ/ARR (1)	Control in a scrapie transmission study (22)
Romney (22)	F (15)	54 (26–111)	ARQ/ARQ (12), TARQ/TARQ (2), ARR/VRQ (1)	Experimental BSE research flock (20)
	M/N (7)	63 (28–86)	ARQ/ARQ (7)	
Suffolk (40)	F (17)	38 (17–87)	ARQ/ARQ (12), ARQ/TARQ (5)	Experimental BSE research flock (20)
	M/N (23)	45 (17–86)	ARQ/ARQ (15), ARQ/TARQ (8)	
Suffolk (7)	F (2)	89 (both)	ARQ/ARQ (7)	Controls for ovine BSE attack rate study (23)
	M/N (5)	88 (88–89)		
Cheviot (21)	F (21)	73 (28–78)	VRQ/VRQ (19), AHQ/AHQ (1), ARR/ARR (1)	Classical scrapie-free flock (19)
Suffolk (20)	F (13)	47 (32–54)	ARQ/ARQ (8), ARR/ARR (5)	Classical scrapie-free flock (19)
	M (7)	42 (29–54)	ARQ/TARQ (3), ARR/ARR (2), ARQ/ARQ (2)	
Poll Dorset (4)	F (2)	71, 72	ARR/ARR (1), ARQ/VRQ (1)	Derived from classical scrapie-free flock (19), intracerebrally inoculated with TSE-free bovine brain homogenate
	M/N (2)	72, 72	ARR/ARR (1), VRQ/VRQ (1)	

*^a^Sex: F, female; M, male; M/N, male neutered*.

*^b^*PRNP*: shown are polymorphisms at codon 112 (*T*hreonine, wild-type Methionine not listed), 136 (*A*lanine or *V*aline), 154 (A*R*ginine or *H*istidine), and 171 (Glutamine *Q* or A*R*ginine)*.

### Test Population

#### Sheep with Scrapie-Like or Other Neurological Signs or Exposed to Scrapie with a Negative TSE Test Result (Test Group 1)

These were 30 sheep (16 females, 2 males, and 12 neutered males) of six different pure- or crossbreeds with a median age of 61 months (range 20–133). They were experimentally challenged with TSE strains or exposed to TSE-affected sheep. Some presented with clinical signs suggestive of a TSE (25 sheep), while others were clinically healthy but raised in a scrapie-affected flock (three sheep) or presented with neurological signs suggestive of a space-occupying lesion confirmed as brain abscess at *postmortem* (see Table 2 for details). The TSE status was based on postmortem tests (for tests, see below). All animals in this group tested negative for TSEs on examination of the brain.

**Table 2:** **Details and origin of sheep negative for TSE on postmortem tests of the brain**.

Exposed or inoculated TSE strain (*N*)	Median age (range) (months)	Sex^a^ (*N*)	Breed (*N*)	*PRNP* (*N*)^b^	Source
C-type BSE^c^ (13)	60 (30–87)	F (8), M/N (5)	Suffolk (7), Romney (4), Cheviot (2)	*ARQ/ARQ* (5), *ARR/ARQ* (3), *ARQ/TARQ* (3), *VRQ/VRQ* (2)	Experimental BSE research flock (20), BSE attack rate study (23)
H-type BSE (1)	66	M/N (1)	Cheviot (1)	*AFRQ/AFRQ* (1)	Atypical BSE transmission study^d^
L-type BSE (1)	33	M/N (1)	Cheviot (1)	*AFRQ/AFRQ* (1)	Atypical BSE transmission study^d^
Classical scrapie (11)	59 (20–133)	F (8), M (1), M/N (2)	Poll Dorset pure- and crossbred (4), Suffolk pure- and crossbred (3), Charollais × (1), Cheviot (3)	*ARR/ARR* (2), *ARR/VRQ* (2), *ARQ/VRQ* (2)^e^, *ARQ/ARQ* (2), *AFRQ/ARQ* (1), *ARQ/TARQ* (1), *VRQ/VRQ* (1)	Natural scrapie flock (24), scrapie milk transmission study (25)
Atypical scrapie (2)	60, 68	M/N (2)	Cheviot (2)	*AFRQ/AFRQ* (2)	Atypical BSE transmission study^d^
Brain abscess (2)	64, 72	M (1), M/N (1)	Cheviot (2)	*AFRQ/AFRQ* (1), *VRQ/VRQ* (1)	Classical scrapie-free flock (19)

*^a^Sex: F, female; M, male; M/N, male neutered*.

*^b^*PRNP*: shown are polymorphisms at codon 112 (*T*hreonine, wild-type *M*ethionine not listed), 136 (*A*lanine or *V*aline), 141 (Phenylalanine *F*, wild-type *L*eucine not listed), 154 (A*R*ginine or *H*istidine), and 171 (Glutamine *Q* or A*R*ginine)*.

*^c^Two sheep orally challenged with 5 g of BSE brainstem, others are the first or second generation of orally challenged ewes*.

*^d^Atypical BSE transmission study: sheep of different PRNP genotypes were intracerebrally inoculated with either L-type or H-type BSE brain homogenate, controls were inoculated with TSE-free bovine brain homogenate*.

*^e^Includes a sheep with detectable PrP^Sc^ in the lymphoreticular system only*.

#### Sheep with Clinical Signs of Disease and a Brain-Positive TSE Test Result (Test Group 2)

A total of 182 sheep (94 females and 88 neutered males) of six different pure- or crossbreeds and various *PRNP* genotypes with a median age of 25 months (range 18–139) were examined, which were inoculated/challenged with or exposed to CS, atypical scrapie, classical BSE, or atypical (L-type) BSE. More details of the sheep and their study origin are given in Table 3.

**Table 3:** **Details and origin of sheep positive for TSE on postmortem tests of the brain**.

Exposed or inoculated TSE strain (*N*)	Median age (range) (months)	Sex^a^ (*N*)	Breed (*N*)	*PRNP* (*N*)^b^	Source
C-type BSE (31)	24 (18–139)	F (13), M/N (18)	Cheviot (24), Suffolk (7)	*AHQ/AHQ* (20), *ARQ/ARQ* (7), *AFRQ/ARQ* (1), *VRQ/VRQ* (1), *ARQ/TARQ* (2)	Experimental BSE research flock (20) and tissue production study (26)
L-type BSE (23)	36 (21–59)	F (9), M/N (14)	Poll Dorset (9), Cheviot (14)	*AFRQ/AFRQ* (8), *ARQ/ARQ* (4), *ARQ/VRQ* (9), *VRQ/VRQ* (2)	Atypical BSE transmission experiment^c^
Classical scrapie (112)	24 (18–93)	F (68), M/N (44)	Poll Dorset pure- and crossbred (68), Cheviot (20), Swaledale (14), Highlander (6), Suffolk pure- and crossbred (2), Texel (2)	*VRQ/VRQ* (83), *ARQ/VRQ* (12), *ARQ/ARQ* (9), *AFRQ/ARQ* (3), *AFRQ/AFRQ* (1), *ARQ/ARH* (2), *ARR/ARQ* (1), *ARR/VRQ* (1)	Natural scrapie flock (24), scrapie field suspects, scrapie pathogenesis study (22), scrapie milk transmission study (25)
Atypical scrapie (16)	36 (18–96)	F (4), M/N (12)	Cheviot (15), Poll Dorset (1)	*AHQ/AHQ* (14), *AFRQ/VRQ* (1), *VRQ/VRQ* (1)	Atypical scrapie transmission study, tissue production study (27, 28)

*^a^Sex: F = female, M = male, M/N = male neutered*.

*^b^*PRNP*: shown are polymorphisms at codon 112 (*T*hreonine, wild-type *M*ethionine not listed), codon 136 (*A*lanine or *V*aline), 141 (Phenylalanine *F*, wild-type *L*eucine not listed), 154 (A*R*ginine or *H*istidine), and 171 (Glutamine *Q*, A*R*ginine or *H*istidine)*.

*^c^Atypical BSE transmission experiment: sheep of different PRNP genotypes were intracerebrally inoculated with bovine or sheep-passaged L-type BSE brain homogenate*.

The majority of the test population (84% of the TSE-positive and 57% of the TSE-negative sheep), but only 31% of the controls, were examined on the day of cull; recordings were obtained in the remaining sheep at a median time of 6 days prior to cull (range 1–385 days) in the controls, 7 days (1–15) in the scrapie-negative, and 2 days (1–9) in the scrapie-positive test population.

### BAEP Recordings

For the recording, the sheep’s head was restrained with a halter, which also enabled attachment of electrodes. The ears were cleaned with cotton swabs to remove any excess cerumen. Sedatives were not used. BAEPs were recorded with the Nicolet VikingQuest EMG portable unit (Natus, Middleton, WI, USA) at 75 and 95 dB normal hearing level (nHL) using insert foam ear tips (13 mm diameter, supplied by Natus). The settings and electrode positions were similar to a previously published study (6): recordings were taken with disposable stainless steel electroencephalogram needle electrodes (12 mm × 0.40 mm with 2.5 m cable, supplied by Natus) that were inserted subdermally at the central poll (Cz; +) and the ipsilateral ear base (A1 or A2; −). The ground electrode was placed central between the medial canthus of both eyes. BAEPs were averages of 1,000 responses to clicks at a rate of 11.4 Hz of alternating polarity, with a recording bandwidth of 150–3,000 Hz. A masking noise of 60 dB nHL was used for the non-test ear. Recordings were only repeated for those ears where the waveform was poor or considered abnormal to confirm the initial recording.

For each waveform, peaks I–V were determined to calculate I–III, I–V, and III–V interpeak latencies (IPL) and V/I amplitude ratio based on the recommended standards by the International Federation of Clinical Neurophysiology (15). The data were checked for normal distribution prior to establishing a reference range and statistical analysis using GraphPad Prism 6 (GraphPad Software, Inc., La Jolla, CA, USA) and Statistica 12 (Statsoft, Tulsa, OK, USA). Absolute latencies, IPL, and V/I amplitude ratio values were tested for normal distribution by the D’Agostino and Pearson omnibus normality test (accepted if *P* > 0.05) and comparison between groups was made using the Student’s *t* or Mann–Whitney test if data were not normally distributed. Proportions of animals were compared using the Fisher’s exact test or Chi-square test where appropriate. When data derived from more than two groups were compared, Bonferroni’s correction for multiple comparisons was employed.

### Postmortem Examination and TSE Diagnostic Tests

The ear canals of culled sheep were checked for the presence of cerumen. The postmortem TSE test protocol varied depending on the study and tests were one or a combination of tests, such as immunohistochemistry as described for ovine BSE, classical and atypical scrapie (28–30), and rapid postmortem test [TeSeE ELISA or TeSeE Western blot (Bio-Rad Laboratories Ltd., Hemel Hempstead, UK)] (31), according to the manufacturer’s instructions. Clinically healthy sheep from the CS-free flock were only tested by the ELISA; the TSE diagnostic test in the other sheep always included immunohistochemistry.

Lymphoreticular tissues were also examined by immunohistochemistry from all sheep except for sheep from the CS-free flock and reported field suspects.

### Assessment of Auditory Pathways and Clinic–Pathological Relationship

To assess whether electrophysiological abnormalities in the auditory pathways were associated with different levels of PrP^Sc^ immunolabelling and vacuolation, brain sections of the auditory system (32) were selected, retrospectively, from a subset of animals from test group 2 for a more detailed neuropathological examination after the BAEP data had been analyzed. The neuroanatomical areas examined were the auditory cortex, medial geniculate nucleus, caudal (inferior) colliculus, lateral lemniscus, trapezoid body, dorsal cochlear nucleus, and dorsal olivary nucleus. Examinations were carried out blind by the pathologist and included sections from 12 sheep with normal and 12 sheep with abnormal BAEP results. Tissue selection was influenced by availability of tissue (e.g., only one-half of the brain, or less, was fixed depending on the individual study postmortem protocols and tissue requirements). Vacuolation of the gray matter neuropil was scored in hematoxylin–eosin stained tissue sections from 0 (absent) to 5 (very numerous and confluent) as described previously (33). Tissue sections were immunolabelled with rat monoclonal antibody R145 (APHA Weybridge, Addlestone, UK) for simplified PrP^Sc^ scoring on a scale from 0 (none) to 3 (striking) to distinguish between four different PrP^Sc^ patterns: (i) intraneuronal, (ii) intraglial (intramicroglial and intrastrocytic types combined), (iii) extracellular glia-associated (subpial, subependymal, perivascular, stellate, and perivacuolar types combined), and (iv) extracellular neuropil-associated (diffuse particulate, coalescing, perineuronal, and linear types combined) (34).

The Wilcoxon matched pairs signed-rank test was used to compare pathological data from sheep with confirmed TSE, which presented with either normal or abnormal BAEPs, matched by TSE strain and approximate age. For simplification, the PrP^Sc^ and vacuolation scores, respectively, for each neuroanatomical area were added to obtain a total score for PrP^Sc^ immunolabelling and vacuolation in the auditory pathways. However, as not all sheep had brain sections available that included all the neuroanatomical areas mentioned above, total scores were compared pair-wise: (a) for areas that were scored in every animal (so total score was for identical areas in all sheep), (b) for areas that were scored in each pair (so some pairs had all areas compared and others only those that had been examined in both sheep of a pair), and (c) for all areas only in those sheep where all areas were actually represented in the tissue sections (so total scores were for all areas but only in certain pairs).

## Results

### Control Population

Complete blockage of the ear canal with cerumen was observed in seven sheep (5.0%), which was bilateral in two and unilateral in five sheep and resulted in complete absence of peaks I–V in two sheep. Recordings from the ears with blocked ear canals were not used to establish a reference range.

In the recordings from ears without blocked ear canals (139 sheep), peaks II and III could not be identified in one (0.72%; unilateral) and eight (6.0%; bilateral in two) sheep, respectively at, 95 dB nHL. At 75 dB nHL, identification was not possible for peak I in 8 sheep (5.8%; bilateral in 1), for peak II in 11 (7.9%; bilateral in 1), for peak III in 45 (32.4%; bilateral in 9), and for peak V in 28 (20.1%; bilateral in 4) sheep. Peaks were not at all identifiable in seven sheep (5.0%; bilateral in one) at 75 dB nHL.

The reference ranges were calculated from all data (left and right ear) recorded at 75 dB and 95 dB nHL, regardless of gender. IPLs and amplitude ratio were not significantly different (*P* > 0.05, *t*-test, or Mann–Whitney test if data were not normally distributed) to justify determining reference ranges for (neutered) male and female sheep separately. In addition, although significant differences between (neutered) male and female sheep were found in the absolute latencies I (*P* = 0.02, Mann–Whitney test) and II (*P* = 0.006, Mann–Whitney test) at 95 dB nHL and for I (*P* = 0.04, Mann–Whitney test), II (*P* = 0.004, unpaired *t*-test), and V (*P* = 0.02, Mann–Whitney test) at 75 dB nHL, with mean or median latencies generally shorter in female sheep, the reference ranges did not necessarily vary. For example, the same reference range was obtained for latency I at 75 dB nHL in female and male (neutered) sheep. The reference range is shown in Table 4.

**Table 4:** **Reference range of BAEP parameters at 95 and 75 dB nHL derived from the control population**.

	95 dB nHL	75 dB nHL
Lat I (ms) (*N*)	1.00–1.25 (273)^a^	1.15–1.45 (264)^a^
Lat II (ms) (*N*)	1.80–2.10 (272)^a^	1.93–2.25 (260)
Lat III (ms) (*N*)	2.50–3.05 (263)^a^	2.61–3.28 (219)^a^
Lat V (ms) (*N*)	3.35–3.87 (273)	3.45–4.10 (242)
IPL I–III (ms) (*N*)	1.40–1.90 (263)^a^	1.34–1.95 (219)^a^
IPL III–V (ms) (*N*)	0.54–1.08 (263)	0.55–1.15 (210)^a^
IPL I–V (ms) (*N*)	2.24–2.73 (273)	2.19–2.78 (242)
V/I amplitude ratio (%) (*N*)	8.89–127.30 (273)^a^	7.62–200.80 (241)^a^

*N is the number of recordings used to determine the range*.

*^a^Calculated as the interval defined by the 2.5th and 97.5th percentiles of the empirical distribution of the measurements because data not normally distributed; otherwise calculated as mean ± 1.96 × SD*.

### Test Population

Abnormalities included prolonged I–III, III–V, and I–V intervals at 95 dB or 75 dB nHL, which could be unilateral or bilateral, unilateral reduced V/I amplitude ratio at 95 dB or 75 dB nHL or unilateral or bilateral absence of waves I to V or V only at 95 dB without evidence of blockage of the ear canal.

#### Sheep with Scrapie-Like or Other Neurological Signs or Exposed to Scrapie with a Negative TSE Test Result (Test Group 1)

Complete blockage of one ear canal with cerumen was noticed in three sheep (10%; two kept in the natural scrapie flock, one intracerebrally inoculated with H-type BSE). While the two scrapie-exposed sheep had detectable peaks in the affected site, which were within the reference range, no peaks were identifiable in the H-type BSE-inoculated sheep, which presented with side-to-side movements of the head and circling when blindfolded, suggestive of an impairment of the vestibular system. Recordings in the contralateral ear were within the reference range.

Recordings that were outside the reference range at 95 dB nHL or 75 dB nHL were seen in seven (23%) sheep, of which three (43%) had bilateral abnormalities. Five of the seven (71%) sheep presented with TSE-like signs prior to cull, one had signs of a space-occupying brain lesion, and one was exposed to CS but clinically healthy and also scrapie-negative on examination of lymphoid tissue. See Table 5 for more details about these sheep.

**Table 5:** **Abnormal BAEP recordings in test group 1**.

Case	Exposure to	Abnormality	*PRNP* genotype	Clinical status
N1	Classical scrapie	Prolonged IPL III–V at 95 dB nHL, unilateral	*ARQ/VRQ*	Clinically healthy
N2	C-type BSE	Prolonged IPL I–III at 95 dB nHL and I–V at 95 and 75 dB nHL and reduced V/I amplitude ratio at 75 dB nHL in one ear; prolonged IPL I–III at 95 dB nHL in the other ear	*ARQ/ARQ*	TSE-like signs
N3	C-type BSE	Reduced V/I amplitude ratio at 95 and 75 dB nHL in one ear; prolonged IPLs III–V and I–V at 75 dB nHL in the other ear	*ARQ/ARQ*	TSE-like signs
N4	C-type BSE	Reduced V/I amplitude ratio at 95 dB nHL, unilateral	*ARQ/TARQ*	TSE-like signs
N5	C-type BSE	Prolonged IPLs I–III and I–V at 95 dB nHL, prolonged IPLs III–V and I–V at 75 dB nHL in one ear; only peak I in the other ear at 95 and no peaks at 75 dB nHL	*VRQ/VRQ*	Signs of space-occupying brain lesion
N6	L-type BSE	No peak V identifiable at 95 dB nHL, unilateral	*AFRQ/AFRQ*	TSE-like signs
N7	H-type BSE	Reduced V/I amplitude ratio at 75 dB nHL, unilateral	*AFRQ/AFRQ*	TSE-like signs

At 95 dB nHL, peak III was not identifiable in four sheep (13%; bilateral in one), which included the two sheep where peak V was also not identifiable. At 75 dB nHL, wave III was undetectable in one (3%) and wave V in three (10%) sheep, and one sheep (3%) had no identifiable peaks at all.

Only one sheep, which was a clinical suspect from a CS flock, had detectable PrP^Sc^ in lymphoid tissue but the BAEPs were within the normal limit. BAEPs were also normal in a sheep with a brain abscess confined to the right (75%) and left (25%) side of the parietal and temporal cortex, which presented clinically with dullness and asymmetrical signs [drifting/circling to the right and right visual impairment (absent menace response with normal pupillary light reflex)].

#### Sheep with Clinical Signs of Disease and a Positive TSE Test Result in Brain (Test Group 2)

**Figure 1:**
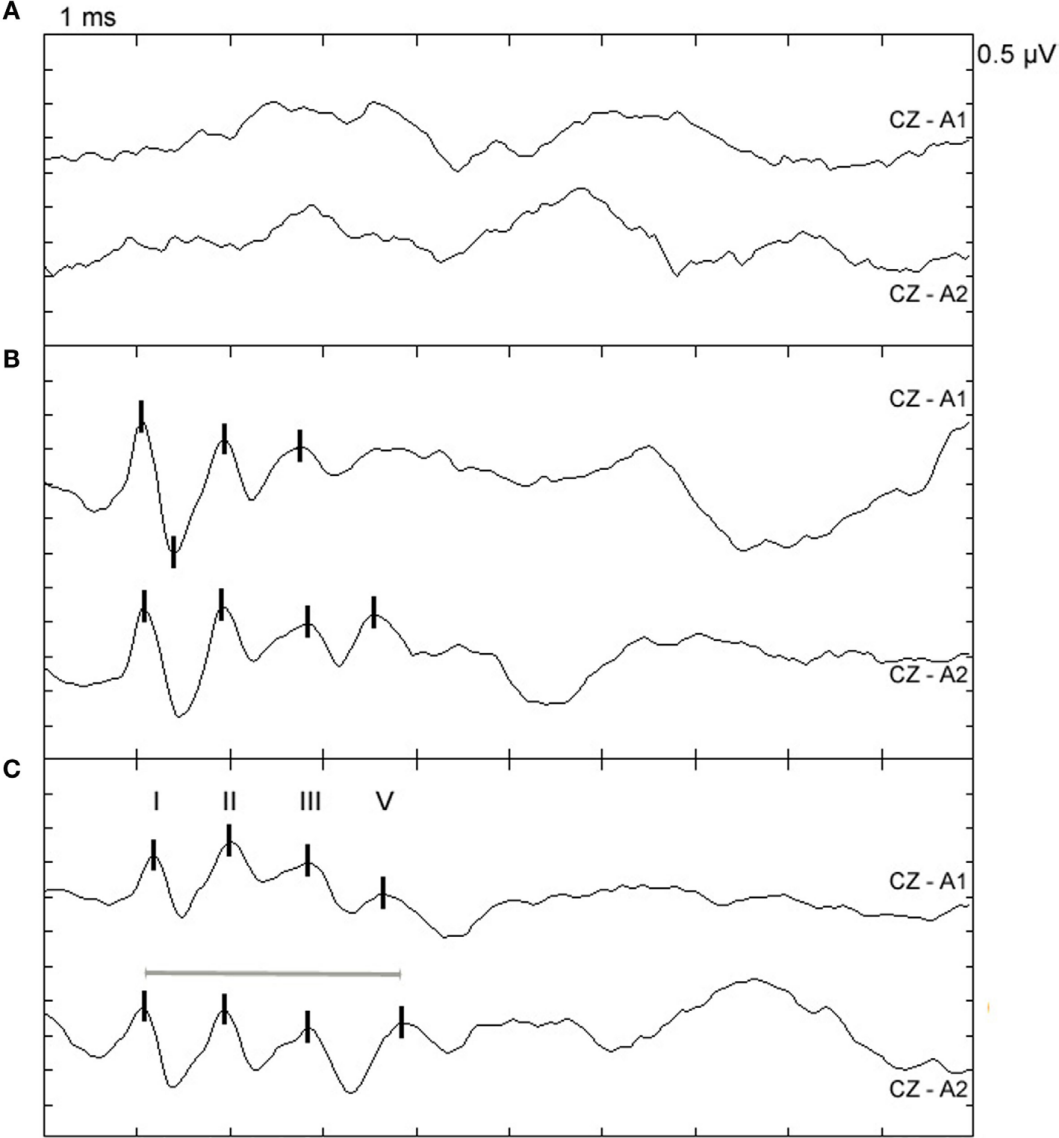
**Examples of BAEP abnormalities in sheep with confirmed TSE**. Top (CZ-A1): left ear recording; bottom (CZ-A2): right ear recording at 95 dB nHL; peaks I, II, III, and V are marked with vertical lines. **(A)** BAEP recording from a 69-month-old *VRQ/VRQ* Poll Dorset ewe naturally infected with classical scrapie. Peaks cannot be clearly identified but both ear canals were completely blocked by cerumen, which may have caused conductive hearing loss. **(B)** BAEP recording from a 35-month-old *AHQ/AHQ* Cheviot wether intracerebrally infected with atypical scrapie (case AS2). Peak V cannot be clearly identified in the recording from the left ear. **(C)** BAEP recording from a 23-month-old *VRQ/VRQ* Poll Dorset × Friesland wether naturally infected with classical scrapie (case CS16). The I–V IPL is prolonged in the recording from the right ear indicated by the gray line.

The ear canal was blocked with cerumen in 26 sheep (14%), which was bilateral in 3 sheep. This resulted in a complete absence of peaks I–V at 95 dB nHL on the same side as the blockage in 18 of the 26 sheep (69%; 1 bilateral; see example in Figure 1A); in the other eight sheep, one or more peaks were present and, for those where IPLs or V/I amplitude ratio could be calculated, IPLs or V/I amplitude ratio were within the normal range.

Recordings were evaluated from all sheep with no blocked ear canals (156 sheep) and from the contralateral ear of sheep with one blocked ear canal (23 sheep). Recordings that were outside the IPL or V/I amplitude ratio reference range at 95 or 75 dB nHL or that had no identifiable wave V at 95 db nHL were found in 42 of the 179 sheep (23%), of which 12 (29%) had bilateral abnormalities. Of these 42 sheep, 3 had atypical scrapie (19% of all evaluated cases with this TSE type), 23 had CS (21%), 7 had L-type BSE (30%), and 9 had classical BSE (29%;), and the proportion of animals with BAEP abnormalities in the four groups was statistically not significant (*P* = 0.6, Chi-square test). See Table 6 for more details about these sheep and Figures 1B,C for examples of observed abnormalities.

**Table 6:** **Abnormal BAEP recordings in test group 2**.

Abnormality (*N*)	Sound level (dB nHL)	Case with TSE strain prefix (*PRNP* genotype)
Reduced V/I amplitude ratio at 95 dB nHL unilateral (3)	95	CB4 (*AHQ/AHQ*)
		LB7 (ARQ/VRQ), LB8^a^ (*AFRQ/AFRQ*)
Reduced V/I amplitude ratio at 75 dB nHL unilateral (2)	75	CS9, CS10 (*VRQ/VRQ*)
Missing waves I–V bilateral (2)	95	AS1 (*AHQ/AHQ*)
		CB11 (*ARQ/ARQ*)
Missing wave V bilateral (4)	95	CS8 (*VRQ/VRQ*), CS11 (*ARH/ARQ*)
		CB12 (*AHQ/AHQ*)
		LB4^a^ (*AFRQ/AFRQ*)
Missing waves I–V unilateral (6)	95	CS6^a^, CS12, CS13 (*VRQ/VRQ*)
		CB13 (*ARQ/TARQ*)
		LB2, LB4^a^ (*AFRQ/AFRQ*)
Missing wave V unilateral (7)	95	AS2, AS3 (*AHQ/AHQ*)
		CS2^a^ (*ARQ/VRQ*), CS4^b^ (*ARQ/ARQ*), CS6^a^ (*VRQ/VRQ*)
		CB6 (*AHQ/AHQ*)
		LB9^b^ (*ARQ/VRQ*)
Prolonged I–III unilateral (5)	95	CS14^b,a^, CS15^a^ (*VRQ/VRQ*)
		CB2^a^ (*AHQ/AHQ*)
	75	CS14^b,a^, CS15^a^ (*VRQ/VRQ*)
Prolonged I–V unilateral (9)	95	CS2^a^ (*ARQ/VRQ*), CS16, CS17, CS18^b^ (*VRQ/VRQ*), CS19 (*ARQ/ARQ*), CS20 (*ARH/ARQ*)
	75	LB8^a^ (*AFRQ/AFRQ*)
		CB2^a^ (*AHQ/AHQ*), CB10^a^ (*ARQ/ARQ*)
Prolonged I–V bilateral (4)	95	CS21 (*VRQ/VRQ*), CS22 (*ARQ/VRQ*)
		CB2^a^ (*AHQ/AHQ*), CB10^a^ (*ARQ/ARQ*)
Prolonged III–V unilateral (11)	95	CS23 (*ARQ/ARQ*), CS24, CS25, CS26 (*VRQ/VRQ*), CS2^a^, CS27 (*ARQ/VRQ*)
		CB8^c^ (*ARQ/ARQ*), CB14 (*AHQ/AHQ*)
	75	LB6 (*AFRQ/AFRQ*), LB10^b^ (*ARQ/ARQ*)
		CB10^a^ (*ARQ/ARQ*)
Prolonged III–V bilateral (1)	95	CB10^a^ (*ARQ/ARQ*)

*TSE strains are indicated in the case number prefix: AS, atypical scrapie; CS, classical scrapie; CB, classical BSE; LB, L-type BSE*.

*^a^Other abnormality present on same or other side*.

*^b^Other ear blocked with wax*.

*^c^Natural infection (in experimental flock)*.

Grouped by genotype, abnormalities were seen in 15 *VRQ/VRQ* sheep (18% of sheep with this genotype, excluding 3 sheep with bilaterally blocked ear canals), 4 *AFRQ/AFRQ* (44%), 7 *ARQ/ARQ* (35%), 8 *AHQ/AHQ* (24%), 2 *ARH/ARQ* (100%), 5 *ARQ/VRQ* (23%), and 1 *TARQ/ARQ* sheep (50%), but none in sheep with genotypes *ARR/VRQ, ARR/ARQ*, and *AFRQ/ALRQ*. The numbers for some genotypes were too small for comparison but the proportion of sheep of the genotypes *VRQ/VRQ, AFRQ/AFRQ, ARQ/ARQ, AHQ/AHQ*, and ARQ/VRQ was not significantly different (*P* = 0.3, Chi-square test).

Wave identification at 95 dB nHL was not possible for wave III in 13 sheep (7%, unilateral) and both waves II and III in 1 sheep (0.6%, unilateral), even though the other peaks were present and the ear canals were not blocked with cerumen.

At 75 dB nHL, wave I could not be identified in 2 sheep (1%), wave II in 1 sheep (0.6%, bilateral), wave III in 25 sheep (14%; bilateral in three), and wave V in 22 sheep (12%; bilateral in one) despite the presence of the other waves. Two waves were missing in 25 sheep (14%; bilateral in five), three missing in 8 sheep (4%), and none of the waves were identifiable in 26 sheep (15%; bilateral in two sheep) with no evidence of blockage of the ear canals.

### Assessment of Auditory Pathways and Clinic–Pathological Relations

There was wide variation in the vacuolation and total PrP^Sc^ scores of the examined neuroanatomical areas between individual animals in test group 2 ranging from 0 to 3 (median: 0.2) for vacuolation and 0 to 7.5 (median: 2) for PrP^Sc^ accumulation. There was a tendency for the more rostral areas to have lower PrP^Sc^ scores (e.g., auditory cortex median: 0, medial geniculate nucleus median: 1.05) than more caudal areas (e.g., dorsal cochlear nucleus median: 2.7, dorsal olivary nucleus median: 3.5), but this was not seen for vacuolation scores (auditory cortex median: 0, medial geniculate nucleus median: 0.1, dorsal cochlear nucleus median: 0.2, dorsal olivary nucleus median: 0; highest medians of 0.5 in lateral lemniscus and trapezoid body).

**Figure 2:**
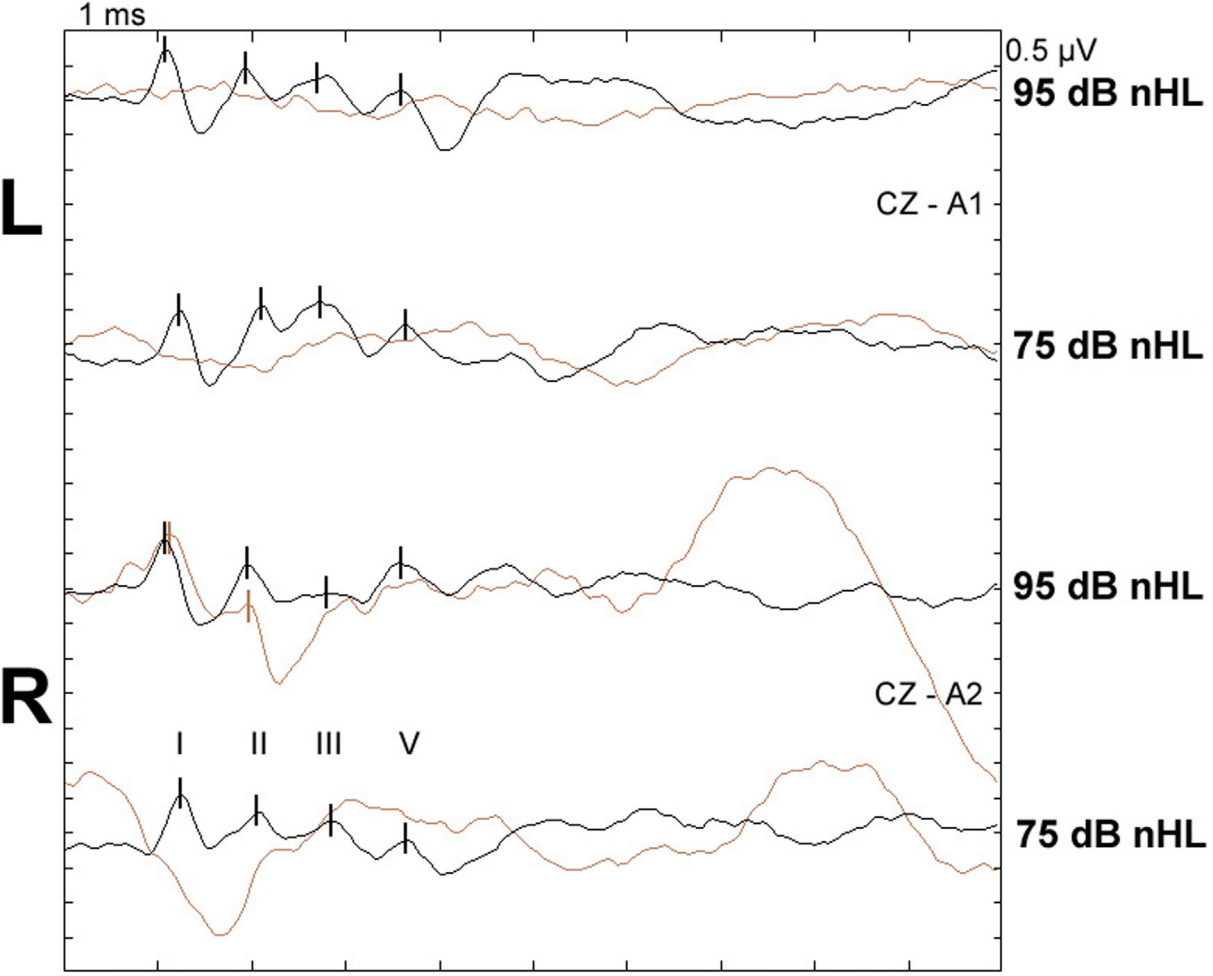
**BAEPs recorded from two scrapie-infected sheep with and without abnormalities**. Top two waveform pairs (CZ-A1): left ear recording, bottom two waveform pairs (CZ-A2): right ear recording at 95 and 75 dB nHL, respectively; peaks I, II, III, and V are marked with vertical lines. Black: normal recording from classical scrapie case CS5 (24-month-old *VRQ/VRQ* Poll Dorset × Friesland ewe; total PrP^Sc^ and vacuolation scores in examined auditory pathways were 12.4 and 2.6, respectively). Brown: recording from classical scrapie case CS6 (22-month-old *VRQ/VRQ* Poll Dorset × Friesland wether) with missing waves I–V left and missing wave V right at 95 dB nHL and no identifiable waves as 75 dB nHL; total PrP^Sc^ and vacuolation scores were 0.4 and 0.6, respectively; ear canals were not blocked by cerumen.

**Figure 3:**
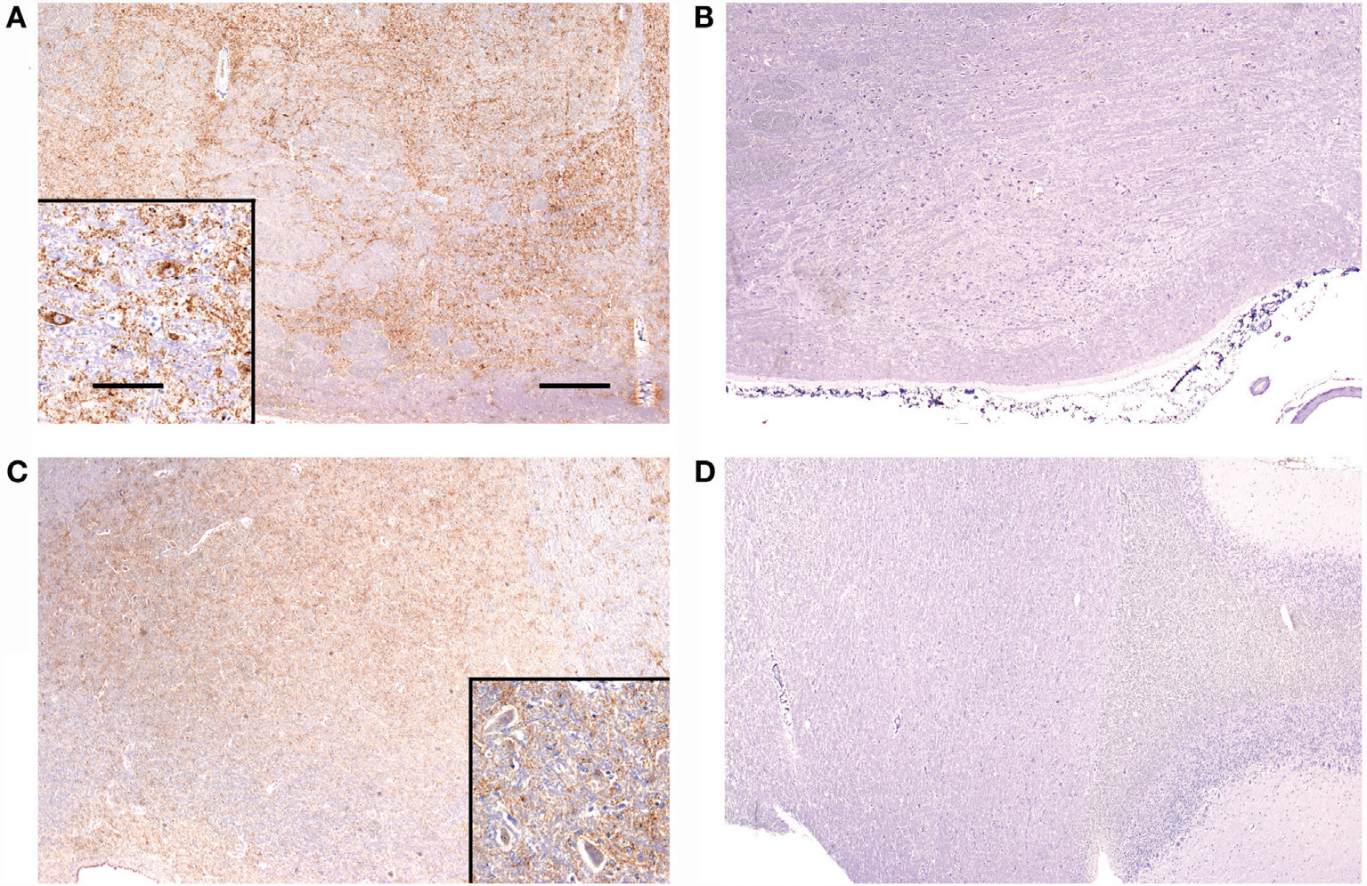
**Immunolabelling of the nucleus of the trapezoid body and dorsal cochlear nucleus from sheep with and without BAEP abnormalities**. PrP^Sc^ immunolabelling is marked in the nucleus of the trapezoid body (overall PrP^Sc^ score 7) from classical BSE case CB4 **(A)**, which presented with BAEP abnormalities, whereas immunolabelling is absent (overall PrP^Sc^ score 0) in CB3 **(B)**, which had a BAEP within normal limits. Classical scrapie case CS4 **(C)** has marked immunolabelling (overall PrP^Sc^ score 6) in the dorsal cochlear nucleus, contrary to classical scrapie case CS6 **(D)** with no detectable PrP^Sc^ (overall PrP^Sc^ score 0), even though both presented with abnormal BAEPs. Antibody R145. Scale bars are 400 μm for **(A–D)** and 40 μm for insets in **(A,C)**.

The total scores of PrP^Sc^ accumulation and vacuolation in auditory pathways in sheep with and without BAEP abnormalities are displayed in Table 7. There were no significant differences in the total PrP^Sc^ or vacuolation scores in the auditory pathways between TSE sheep with and without abnormal BAEPs (*P* > 0.05, Wilcoxon matched pairs signed-rank test), when all cases were compared. However, the data suggested that there was a relationship between PrP^Sc^ scores and BAEPs abnormality depending on the TSE strain: unlike in CS (see Figure 2 as example of two CS sheep with and without BAEP abnormalities), PrP^Sc^ scores were consistently higher in classical and L-type BSE-infected sheep with BAEP abnormalities than in those without, although this difference was only significant if C-BSE and L-type BSE-infected sheep were combined (*P* = 0.03 for areas that were scored in every animal and *P* = 0.02 for areas that were scored in each pair, respectively, Wilcoxon matched pairs signed-rank test). Figure 3 provides examples of PrP^Sc^ accumulation or absence in the nucleus of the trapezoid body and dorsal cochlear nucleus in classical BSE and CS-infected sheep with and without abnormal BAEPs. This effect was not seen when the vacuolation scores were compared.

**Table 7:** **Comparison of total scores of PrP^Sc^ accumulation and vacuolation in auditory pathways in sheep with and without BAEP abnormalities**.

	BAEP		BAEP		BAEP	
PrP^Sc^ accumulation	Normal	Abnormal	Normal	Abnormal	Normal	Abnormal
			
Case comparison	Score a		Score b		Score c	
CB1 cf. CB2	3.4	5.5	7.8	14.7		
CB3 cf. CB4	1.4	7.5	2.4			27
CB5 cf. CB6	5.2	7.1	13.2	15.9	19	14.6
CB7 cf. CB8	2.1	3.4	9.5	9.5	12.1	12.1
CB9 cf. CB10	3.9	7	15.9	2.4	27	19
LB1 cf. LB2	13	11.7	17	16.7	14.7	22.7
LB3 cf. LB4	16	18	33.5			37.5
LB5 cf. LB6	9.7	10.7	14.7			22.7
CS1 cf. CS2	0.7	5.9	2.1	12.9	12.4	0.4
CS3 cf. CS4	8.5	9.5	11.5			18.7
CS5 cf. CS6	3.9	0	12.4			0.4
CS7 cf. CS8	3.7	2.5	12.9			6.5
**Vacuolation**						
CB1 cf. CB2	0.7	0.5	1.9	1.5		
CB3 cf. CB4	0.5	0	1.7			2.2
CB5 cf. CB6	0	0	0.5	1.7	2.2	1.2
CB7 cf. CB8	0	0.2	1.5	1.5	1.2	1.2
CB9 cf. CB10	0	0.9	1.5	1.5	2.9	2.9
LB1 cf. LB2	2.5	3	3	3.2	12	8.9
LB3 cf. LB4	2.7	4.5	6.7			9
LB5 cf. LB6	6.5	6	12			8.9
CS1 cf. CS2	0	0	0.4	1	2.6	0.6
CS3 cf. CS4	0.7	0	1.1			2
CS5 cf. CS6	0.7	0	2.6			0.6
CS7 cf. CS8	0	0.2	1.2			1.4

*Compared cases were affected by Classical BSE (CB), L-type BSE (LB), and Classical scrapie (CS)*.

*Score a: total for neuroanatomical areas that were scored in every animal*.

*Score b: total for neuroanatomical areas that were scored in each pair*.

*Score c: total score for all areas only in those sheep where all areas were actually represented in the tissue sections (see Materials and Methods)*.

## Discussion

Transmissible spongiform encephalopathies are neurological diseases that cause behavioral, locomotor, and sensory changes in affected ruminants. While scrapie in sheep may also affect vision, as characterized by an absent menace response (35–37), apparent visual disturbance (19, 38), and altered electroretinograms (39, 40), which have been associated with PrP^Sc^ accumulation in the retina, little is known about the effect of the TSE agent on hearing. Repeated startle responses to hand clapping were found in 29% of 129 sheep naturally infected with scrapie (35), but if no startle response is elicited it is not known whether this is due to loss of hearing. In humans with Creutzfeldt-Jakob disease, hearing loss is a rare event limited to case reports (41, 42) and may be confused with auditory agnosia (43). The assessment of auditory function is generally difficult in animals unless specific electrophysiological equipment is used. BAEPs have been used to determine auditory function in various species, such as cats (44), dogs (45), horses (46), cattle (47), camelids (48), and sheep (49), which require the establishment of a reference range. Several physiological factors affect BAEP values, which include age [decreased wave V amplitudes have been shown in older horses (50)], sex, and breed [most likely due to differently sized brainstems and corresponding skulls, which have been shown to affect latencies (16)]. We were limited in our control population to four breeds, whereas the test population included breeds that were not represented in the controls. Similarly, although the age of the control population was similar to that of the test population, two classical BSE-affected sheep were outliers aged 113 and 139 months, which exceeded the maximum age of 109 months in two control sheep. By combining all control sheep BAEP data, we based our reference range on a large number of sheep (141) but cannot exclude the possibility that more sheep of a particular breed or age might have had values outside the normal range, if we had established values for each breed or age group separately. To establish a reference range based on a large number of sheep, we had to include sheep that were in contact with sheep that subsequently developed BSE. By confirming (i) absence of detectable PrP^Sc^ in both brain and lymphoid tissue by immunohistochemical examination, which is a confirmatory test and gold standard, (ii) absence of clinical signs associated with TSEs, and (iii) given that “natural” BSE transmission in this experimental flock was not very efficient (20), we were confident that these sheep were indeed not affected by a TSE.

Based on the reference range, 23% of TSE-affected sheep had abnormal BAEP values and the proportion of animals with abnormal BAEPs did not appear to be significantly different across all TSE types (atypical and classical BSE and scrapie) and *PRNP* genotypes. This may be unexpected because the distribution and severity of vacuolation and PrP^Sc^ accumulation is affected by genotype (10, 51) and – more importantly – by different TSE strains in sheep (33, 52, 53). In particular, atypical scrapie differs from CS and BSE by its predominantly white matter immunolabelling and its lack of vacuolar changes in the brainstem (11). We did not score vacuolation or PrP^Sc^ accumulation in atypical scrapie cases as part of this study, but prominent immunolabelling was reported to be fairly consistent in areas of the auditory pathways, such as the dorsal cochlear nucleus, trapezoid body, and medial geniculate nucleus, in naturally occurring atypical scrapie (11), which was reproduced in experimental disease (28). Scoring of vacuolation and PrP^Sc^ accumulation in the auditory pathways of the TSE-infected sheep with and without BAEP abnormalities did not provide evidence that abnormalities were associated with differences in vacuolar changes or PrP^Sc^ accumulation. This simply provides more evidence that neither PrP^Sc^ nor vacuolation are responsible for clinical dysfunction. We have previously reported that both TSE markers were poorly associated with clinical signs in ruminants (54, 55). This is in contrast to a neuropathological study in cattle intracerebrally infected with BSE, which suggested that spongiform changes especially in the caudal colliculus may be related to prolonged BAEP latency (56). Limiting the comparison to only neuroanatomical areas that are believed to be the generators of wave I to V or only intraneuronal PrP^Sc^ accumulation, which has previously been described as particularly significant marker in relation to the development of clinical disease (57), did not change the results (data not shown). However, the number of cases examined neuropathologically may have been too small to show a significant effect because there was some indication that sheep with atypical and classical BSE and abnormal BAEPs had higher PrP^Sc^ scores. If PrP^Sc^ was indeed responsible for this change, one would have expected the same for CS but no relationship could be found. Route of inoculation was unlikely to be a contributing factor because only L-type BSE cases were infected intracerebrally, whereas CS cases were naturally infected and classical BSE occurred from a combination of oral and natural (in an experimental flock) infection.

As parts of the BAEPs are attributed to corresponding segments of the auditory pathways, BAEP abnormalities may indicate a lesion in certain parts of the auditory pathway, e.g., increased IPL I–III: ipsilateral lesion of more caudal segments of the brainstem; increased IPL III–V: ipsilateral lesion in more rostral and pontine and midbrain segments; absent wave V or a reduced V/I amplitude ratio: ipsilateral caudal and rostral brainstem lesion; and absent waves I–V: cochlear or acoustic nerve lesion (58, 59). We did not assess whether specific BAEP abnormalities were associated with changes in the corresponding neuroanatomical areas due to the limited number of sheep that were subjected to a more detailed neuropathological examination of the auditory pathways, and not all areas were represented in the examined sections.

Occlusion of the ear canal with cerumen was observed in 36 (10%) of all studied sheep and was believed to be the cause of absent BAEP waves recorded from the same ear. In medical practice, it is recommended to check the external ear canal with an otoscope to assure that no blockage is present (8), but this practice is generally not mentioned in studies of conditions in animals, which have no detectable BAEP waves, so it is not known whether this also occurs in other species with narrow ear canals.

Although measurement of BAEPs is easily accomplished without the requirement for sedation in sheep, any observed abnormalities are not disease-specific. For example, one sheep with a brain abscess in test group 1 also presented with BAEP abnormalities. BAEPs have been rarely recorded from sheep with different neurological diseases, which is most likely due to the poor cost–benefit ratio in farm animals and, thus, only reserved for scientific studies rather than diagnostic purposes. The authors are only aware of two neurological conditions in sheep where BAEPs were previously studied: experimentally induced *Trypanosoma brucei* meningoencephalitis, which revealed no abnormalities (49) and scrapie in experimentally (7) and naturally infected sheep (6), which showed prolonged IPL I–III and III–V and amplitude reductions compared to a small number of controls. We also detected abnormalities in sheep that were confirmed negative by the postmortem tests on brain and lymphoid tissue but were exposed to classical or atypical BSE and presented with clinical signs suggestive of a TSE. The significance of this finding is not known. We have previously reported clinical signs in experimentally infected animals, which did not have TSE-confirmed *postmortem*, and the presence of both clinical signs and BAEP abnormalities in animals exposed to a TSE agent may be even more suggestive of a TSE-related brain disease that is not recognized by the current postmortem tests. However, the BAEP recordings were also abnormal in a clinically healthy sheep, which we included in the scrapie-negative test group because it was raised in a scrapie flock with high scrapie incidence (24). Infectivity studies or more sensitive PrP^Sc^ detection methods using brain material from these cases would be needed to assess whether they may have been affected by a TSE which falls below the current postmortem tests detection thresholds.

## Author Contributions

TK carried out the clinical assessments and recorded the auditory evoked potentials, supported by LP. SC was responsible for genotyping results and MS, LG, and MC for the postmortem test diagnosis. LG also provided the scoring of the auditory pathways. TK analyzed the data and drafted the manuscript. All authors read, contributed to, and approved the final manuscript.

## Conflict of Interest Statement

The authors declare that the research was conducted in the absence of any commercial or financial relationships that could be construed as a potential conflict of interest.
