# Supplementary material for: Abnormalities in Brainstem Auditory Evoked Potentials in Sheep with Transmissible Spongiform Encephalopathies and Lack of a Clear Pathological Relationship
Source: Front Vet Sci. 2016 Aug 2;3:60. doi: 10.3389/fvets.2016.00060 (PMC4969942; doi:10.3389/fvets.2016.00060)
Supplement: Supplementary file 1 [file Table_1.doc]

Supplementary Material

Abnormalities in Brainstem Auditory Evoked Potentials in Some Sheep with Transmissible Spongiform Encephalopathies and Lack of a Clear Pathological Relationship

Timm Konold^*^, Laura J Phelan, Saira Cawthraw, Marion M Simmons, Melanie J Chaplin, Lorenzo González

*** Correspondence:** Timm.Konold@apha.gsi.gov.uk

# Supplementary Table

## Table 1. Neuropathological examination scores in 12 sheep each with normal and abnormal BAEP findings

Sheep are arranged as pairs, i.e. the sum of all PrP^Sc^ or vacuolation (Vac) scores in row 1 was compared with row 2, row 3 with row 4 etc.

The TSE strain is indicated in the case prefix: CB = classical BSE, LB = L-type BSE and CS = classical scrapie.

AC = Auditory Cortex; MGN = Medial geniculate nucleus; CC = Caudal colliculus, LL = Lateral lemniscus, TB = Trapezoid body; DC=Dorsal cochlear nucleus; DON = Dorsal olivary nucleus

N/A = Not available

| **Case** | **AC** | | **MGN** | | **CC** | | **LL** | | **TB** | | **DCN** | | **DON** | | **BAEP** | ***PRNP* GENOTYPE** | **BREED** | **AGE** |
| --- | --- | --- | --- | --- | --- | --- | --- | --- | --- | --- | --- | --- | --- | --- | --- | --- | --- | --- |
|  | **PrP** | **Vac** | **PrP** | **Vac** | **PrP** | **Vac** | **PrP** | **Vac** | **PrP** | **Vac** | **PrP** | **Vac** | **PrP** | **Vac** |  |  |  |  |
| CB1 | 0 | 0 | 1 | 0.2 | 0.9 | 0.5 | 2.1 | 0.5 | 3 | 0 | 1.4 | 0.2 | 2.4 | 0.5 | Normal | *AHQ/AHQ* | Cheviot | 28 m |
| CB2 | 0 | 0 | 2 | 0.5 | 2 | 0.5 | 3.5 | 0.5 | N/A | N/A | 3.7 | 0 | 3.5 | 0 | Abnormal | *AHQ/AHQ* | Cheviot | 30 m |
| CB3 | 0 | 0 | 0.4 | 0.5 | 0.4 | 0.2 | 0.2 | 0.5 | 0 | 0 | 0.4 | 0.5 | 1 | 0 | Normal | *AHQ/AHQ* | Cheviot | 21 m |
| CB4 | 0 | 0 | 2.5 | 0 | 1.5 | 0 | 5 | 1 | 7 | 0.2 | 6 | 1 | 5 | 0 | Abnormal | *AHQ/AHQ* | Cheviot | 18 m |
| CB5 | 0 | 0 | 0.2 | 0 | N/A | N/A | N/A | N/A | 1.5 | 0.5 | 6.5 | 0 | 5 | 0 | Normal | *AHQ/AHQ* | Cheviot | 24 m |
| CB6 | 0 | 0 | 1.1 | 0 | 5.2 | 1.5 | 4 | 0.5 | 3.5 | 1 | 4 | 0.2 | 6 | 0 | Abnormal | *AHQ/AHQ* | Cheviot | 24 m |
| CB7 | 0 | 0 | 0.6 | 0 | 2 | 0 | 2 | 1 | 0.9 | 0 | 2.5 | 0.5 | 1.5 | 0 | Normal | *AHQ/AHQ* | Cheviot | 24 m |
| CB8 | 0 | 0 | 0.4 | 0 | 0 | 0 | 1 | 0.5 | 0.7 | 0 | 7 | 0.5 | 3 | 0.2 | Abnormal | *AHQ/AHQ* | Suffolk | 26 m |
| CB9 | 0.5 | 0 | 1 | 0 | 3.5 | 0 | 0.8 | 0 | 5 | 1 | 2.7 | 0.5 | 2.4 | 0 | Normal | *ARQ/ARQ* | Suffolk | 29 m |
| CB10 | 0.5 | 0.2 | 2 | 0.2 | 1.5 | 0 | 2.5 | 0.5 | 3.5 | 0.5 | 4.5 | 1 | 4.5 | 0.5 | Abnormal | *ARQ/ARQ* | Suffolk | 29 m |
| LB1 | 3.5 | 0.5 | 5 | 2 | N/A | N/A | N/A | N/A | 4 | 0.5 | 4.5 | 1 | 4.5 | 0 | Normal | *AFRQ/AFRQ* | Cheviot | 43 m |
| LB2 | 1.2 | 0.5 | 6 | 2.5 | 5 | 2.5 | 2.5 | 1.5 | 5 | 0.2 | N/A | N/A | 4.5 | 0 | Abnormal | *AFRQ/AFRQ* | Cheviot | 45 m |
| LB3 | 2.5 | 0.2 | 7.5 | 2.5 | 7 | 2 | 5.5 | 1.5 | 5 | 0.5 | 6 | 0 | 6 | 0 | Normal | *AFRQ/AFRQ* | Cheviot | 48 m |
| LB4 | 5 | 1.5 | 7.5 | 3 | 7.5 | 2.5 | 6 | 1.5 | 6 | 0.5 | N/A | N/A | 5.5 | 0 | Abnormal | *AFRQ/AFRQ* | Cheviot | 46 m |
| LB5 | 3 | 3 | 4.5 | 3 | 0.4 | 2.5 | 1.5 | 1 | 1.2 | 1 | 1.9 | 1 | 2.2 | 0.5 | Normal | *AFRQ/AFRQ* | Cheviot | 24 m |
| LB6 | 3.5 | 3 | 5 | 2.5 | 3.5 | 2 | 6 | 0.2 | 1 | 0.5 | 1.5 | 0.2 | 2.2 | 0.5 | Abnormal | *AFRQ/AFRQ* | Cheviot | 24 m |
| CS1 | 0 | 0 | 0 | 0 | 1 | 0.2 | 0.4 | 0.2 | 3.5 | 0.2 | 1 | 0 | 0.7 | 0 | Normal | *ARQ/VRQ* | Vendeen x Poll Dorset | 43 m |
| CS2 | 0 | 0 | 0.4 | 0 | 3 | 0.5 | 4 | 0.5 | N/A | N/A | N/A | N/A | 5.5 | 0 | Abnormal | *ARQ/VRQ* | Highlander | 24 m |
| CS3 | 0 | 0 | 3 | 0.5 | 1.5 | 0.2 | 1.5 | 0.2 | 1.5 | 0.2 | N/A | N/A | 5.5 | 0.2 | Normal | *ARQ/ARQ* | Highlander | 24 m |
| CS4 | 0 | 0 | 2.5 | 0 | 7 | 1.5 | 2.2 | 0.5 | N/A | N/A | 6 | 1 | 7 | 0 | Abnormal | *ARQ/ARQ* | Highlander | 60 m |
| CS5 | 1.5 | 0 | 0.4 | 0.5 | 2 | 0.2 | 2 | 1 | 3.5 | 0.5 | 1 | 0.2 | 2 | 0.2 | Normal | *VRQ/VRQ* | Poll Dorset x Friesland | 24 m |
| CS6 | 0 | 0 | 0 | 0 | 0 | 0.2 | 0 | 0.2 | 0.4 | 0.2 | 0 | 0 | 0 | 0 | Abnormal | *VRQ/VRQ* | Poll Dorset x Friesland | 22 m |
| CS7 | 0 | 0 | 0.2 | 0 | 5 | 0.5 | 2.7 | 0.5 | 1.5 | 0.2 | N/A | N/A | 3.5 | 0 | Normal | *VRQ/VRQ* | Poll Dorset | 20 m |
| CS8 | 0 | 0 | 0 | 0 | 1 | 0.2 | 0.5 | 0.5 | 2.5 | 0.5 | 1 | 0.2 | 2.5 | 0.2 | Abnormal | *VRQ/VRQ* | Poll Dorset | 22 m |
